# Acupressure for insomnia: A protocol for systematic review and meta-analysis

**DOI:** 10.1097/MD.0000000000013180

**Published:** 2018-11-09

**Authors:** Dong-Jie Wu, Hai-Cheng Dong, Tsz-Nga Tang, Shi-Feng Zhu

**Affiliations:** a Graduate School, Zhejiang Chinese Medical University, Hangzhou, Zhejiang; b School of Chinese Medicine, Hong Kong Baptist University, Hong Kong; c Department of Tradition Chinese Medicine, Children's Hospital, Zhejiang University School of Medicine, Hangzhou; d Tongde Hospital of Zhejiang Province, Hangzhou, Zhejiang, China.

**Keywords:** acupressure, insomnia, randomized controlled trial, sleep disorder, systematic review

## Abstract

**Background:**

Insomnia is a public sleep disorder defined as a deficiency of sleep quantity or quality. Acupressure is a low-cost treatment that has potential as an insomnia therapy.

**Methods:**

Four databases will be searched from inception to date. The researchers will screen clinical randomized trials of acupressure and auricular acupuncture for insomnia. The screening of the study, data extraction will be carried out independently by 2 researchers. The specific process will refer to the Cochrane Handbook for Systematic Review.

**Results:**

The results of the study will be published in a scientific journal after peer-review. We integrate the latest study about acupressure for insomnia.

**Conclusion:**

This systematic review will provide evidence for assessing the improvement of acupressure for insomnia.

**Ethics and dissemination:**

The systematic review will be published in a peer-reviewed journal. The review will also be disseminated electronically and in print.

PROSPERO registration: CRD42018104155.

## 1 Introduction

Insomnia is a public sleep disorder defined as deficiency of sleep quantity or quality. The disease is associated with difficulty falling asleep, maintaining sleep, or early morning awakening.^[1,2]^ The incidence of insomnia is significantly higher in women and the old adults among the general population. According to current criteria, approximately 12% to 20% individuals can be diagnosed as insomnia, and 35% to 50% of adult population suffers from the related symptoms.^[3–5]^ The insomnia patients often experience fatigue, weakened cognitive function, depression, and other medical and psychiatric comorbidity. Symptoms of insomnia bring high economic burden to the family and approximately $30 billion to $107 billion was estimated spending on the condition in the united state.^[6,7]^

The American Psychiatric Association recommends 3 main methods containing psychological therapy, pharmacologic therapy, and complementary and alternative treatments to treat insomnia.^[8]^ Brief behavioral treatment for insomnia and cognitive behavioral therapy for insomnia are 2 most effective psychological treatments for insomnia disorders improving sleep quality associated with few side effects.^[9–11]^ Multicomponent behavioral therapy, sleep restriction, stimulus control, and relaxation therapy are other psychotherapy approaches. Hypnotics, such as benzodiazepines, are most common used treatment recommended by American College of Physicians Guideline.^[8]^ The third recommended method is complementary and alternative medicine, including acupuncture and Chinese herbal medicine.^[12,13]^

Traditional Chinese medicine, one of the complementary and alternative medical, increasingly being applied to the treatment of mental illness.^[14–16]^ Utilizing acupuncture to treat psychiatric disorder was first accepted in American and European, and relational research that Chinese herbal medicines could improve mental symptoms have gradually increased. Acupressure and auricular acupuncture are not the mainstream methods of Chinese medicine, but they have advantages in the treatment of insomnia syndrome. Several medical researchers have discovered the value of this traditional therapy in the treatment of insomnia syndrome and have designed clinical trials to verify the reliability and safety of this therapy. In a systematic review, Yeung et al first summarize the methods of traditional Chinese medicine treatment of insomnia, including some uncommon therapy such as auricular acupressure, moxibustion, and reflexology.^[17]^ Acupressure has potential as a low-cost alternative treatment for insomnia, and a pilot randomized controlled trial was conducted in Hong Kong to evaluate the short-term effects of self-administered acupressure.^[18]^ Although the outcome regarding sleep onset latency and wake after sleep onset were not significant between 2 groups, but the adherence to practice was satisfactory.^[18]^ We tried to gather evidence to systematically review the value of this simpler therapy.

## 2 Methods

### 2.1 Registration

This protocol has been registered with the PROSPERO registry of the University of York. The registration number is CRD42018104155.

This systematic review protocol will follow the guidelines of Preferred Reporting Items for Systematic Reviews and Meta-Analyses (PRISMA-P).

### 2.2 Eligibility criteria

We will include patients who suffer from sleep disorders, the interventions study used contain acupressure or auricular acupuncture, the control measures used may be sham needles, western medicine, and other measures.

### 2.3 Search methods for identifying the studies

#### 2.3.1 Electronic sources

We will search for MEDLINE, EMBASE, The Cochrane Library (CENTRAL), and China National Knowledge Infrastructure from the inception to October 2018. The searches will be combined with the medical subject headings (Mesh) and keywords of “Insomnia OR Sleeplessness OR Sleep disorder” AND “Acupressure or acupoint or auricular acupressure.” References such as conference documents, research projects, doctoral and Master's thesis will be manually searched.

#### 2.3.2 Study records

##### 2.3.2.1 Data management selection process and data items

All the articles will be confirmed by 2 independent reviewers according to the eligibility of studies, and the discrepancies will be resolved by consensus. All data will be extracted and collected in a standardized template. In the event that information is absent or unclear, the study investigators will contact with the author.

#### 2.3.3 Data collection process

**Figure 1:**
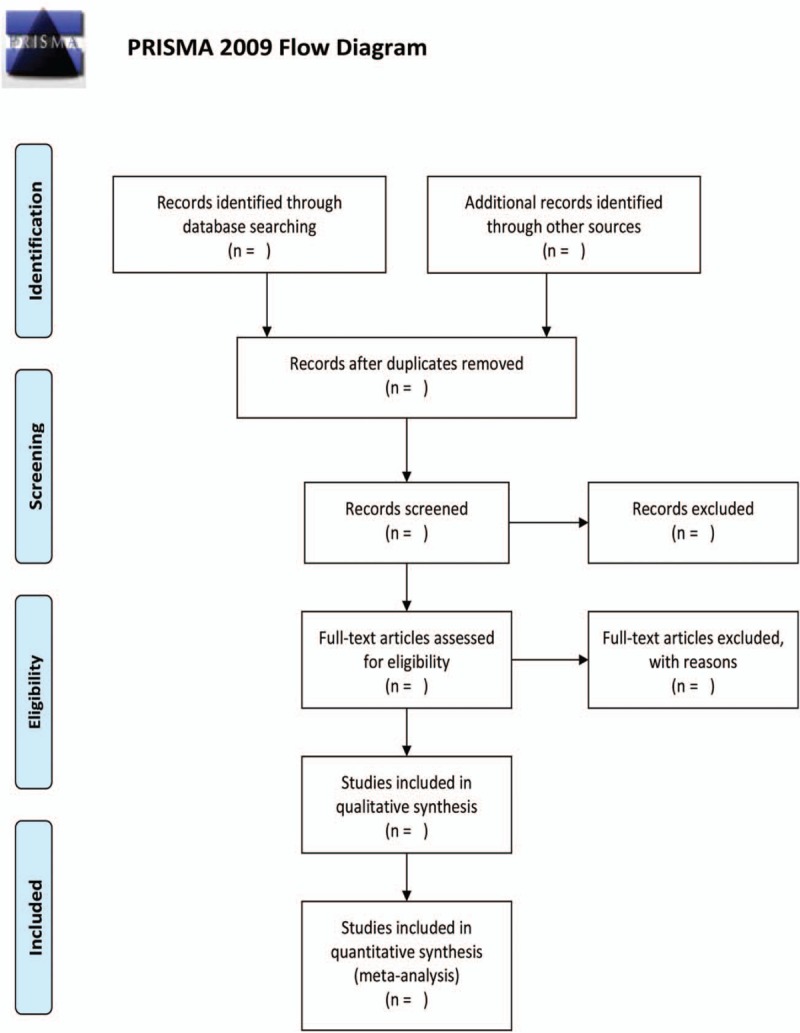
Flow diagram of the study selection process.

Two authors (Hai-Cheng Dong and Tsz-Nga Tang) will independently separate the result information from each investigation according to a standardized data extraction form (Fig. 1). The disagreement will be settled regarding study inclusion by a third reviewer (Dong-Jie Wu) as necessary. We will use the software to remove the duplicate paper and then use manual de-duplication. A template form will be utilized to collect data. The extracted data items will contain the authors, years of publication, study designs, sample sizes, interventions, primary outcome (rates of sleep efficiency), other outcomes (such as sleep onset latency, wake after sleep onset, total sleep time, sleep efficiency), adverse effects, and funding source.

### 2.4 Outcomes

#### 2.4.1 Primary outcomes

Rates of sleep efficiency

#### 2.4.2 Secondary outcomes

Sleep onset latency

Wake after sleep onset

Total sleep time

Adverse events

### 2.5 The risk of bias in individual studies

Two independent reviewers (Hai-Cheng Dong and Tsz-Nga Tang) will separately survey methodological quality utilizing the Cochrane risk of bias tool.^[19]^ The conflicts cannot be settled in the review will search consensus for a third author (Shi-Feng Zhu) as required. Domains need to be evaluated will include: sequence generation; allocation concealment; blinding of participants; blinding of outcome assessment; incomplete outcome data; selective outcome reporting; and other issues.

We will divide each bias domains into unclear, low, and high degrees according to the Cochrane Handbook for Systematic Reviews of Interventions.

### 2.6 Data synthesis

The data displayed as a consistent variable will be utilized to perform meta-analysis by acquiring the mean difference or standardized mean difference (SMD), and to assess the treatment effect with 95% confidence interval (CI). We will use SMD when the different outcome variables are used. For dichotomous outcomes, the risk ratio and 95% CI will be used. Random-effects models will be used for all analyses.

### 2.7 Assessment of heterogeneity

*I*^2^ statistic > 50% indicates that the combined results are heterogeneous (using a random-effects model), and *I*^2^ statistic < 50% indicates that the combined results are homogenous. In addition, the *P* value < .05 indicates that there is a statistical difference between the 2 groups, and the merged diamonds on the graph will not intersect with the straight line. Once intersected, there is no difference between the 2 groups.

### 2.8 Assessment of reporting biases

The Funnel plot produced by Review Manager software is an inverted triangle. The larger the sample size, the more concentrated it is. However, the statistical description cannot be given only by visual inspection. If the 2 sides are symmetrical, there is no obvious publication bias. On both sides, there may be publication bias. The software's Egger test and Begg test gave statistical descriptions, where *P* value > .05 suggests no significant publication bias, and the *P* value < .05 has publication bias. In terms of accuracy, Funnel plot is not as good as Egger test and Begg test, while Begg test is not as sensitive as Egger test. When the 3 results are inconsistent, first give up the Funnel plot. When the Egger test and the Begg test result are opposite, the result of the Egger test will be used as the result.

### 2.9 Analysis of subgroups or subsets

If there are not sufficiently homogeneous in terms of studies, potential subgroup analyses domain will include study design. We will stratify the subgroup by different subdomains.

### 2.10 Confidence in cumulative evidence

The quality of the review will be assessed utilizing the GRADE system.^[19,20]^ According to this grading systems, the result will be appraised as high, moderate, low, and very low quality.^[20]^ We assume that the quality of the evidence at the beginning is the highest, and gradually downgrade the quality evaluation according to the deficiencies of the study.

## 3 Discussion

Insomnia is a highly prevalent problem. Psychotherapy, drug treatment, and complementary and alternative medical treatments are conventional approaches. The purpose of this systematic analysis is to assess the efficacy and safety of acupressure on patients who experiencing insomnia.

It is a potential therapy for insomnia, and several relevant researches have emerged. This systematic review will provide current evidence on the effectiveness and safety of acupressure for insomnia. These findings will provide guidance to clinicians and patients on the use of acupressure for the sleep disorder. The results of this report will be disseminated after peer review and publication.

## Author contributions

Dong-Jie Wu and Shi-Feng Zhu contributed to the conception of the study. The manuscript protocol was drafted by Dong-Jie Wu and was revised by Shi-Feng Zhu. The search strategy was developed by all the authors and performed by Hai-Cheng Dong and Tsz-Nga Tang, who also independently screened the potential studies, extracted data from the included studies, assessed the risk of bias, and completed the data synthesis. Shi-Feng Zhu arbitrated in cases of disagreement and ensured the absence of errors. All authors approved the publication of the protocol.

**Conceptualization:** Dong-Jie Wu, Shi-Feng Zhu.

**Formal analysis:** Hai-Cheng Dong, Tsz-Nga Tang.

**Software:** Hai-Cheng Dong, Tsz-Nga Tang.

**Supervision:** Shi-Feng Zhu.

**Writing – original draft:** Dong-Jie Wu.

**Writing – review & editing:** Dong-Jie Wu.
